# Spatial–Temporal Response of Reactive Oxygen Species and Salicylic Acid Suggest Their Interaction in Pumpkin Rootstock-Induced Chilling Tolerance in Watermelon Plants

**DOI:** 10.3390/antiox10122024

**Published:** 2021-12-20

**Authors:** Fei Cheng, Min Gao, Junyang Lu, Yuan Huang, Zhilong Bie

**Affiliations:** Key Laboratory of Horticultural Plant Biology, Ministry of Education, College of Horticulture and Forestry Sciences, Huazhong Agricultural University, Wuhan 430070, China; feicheng@mail.hzau.edu.cn (F.C.); gaomin7896@126.com (M.G.); jylu@webmail.hzau.edu.cn (J.L.); huangyuan@mail.hzau.edu.cn (Y.H.)

**Keywords:** pumpkin rootstock, grafting, watermelon, salicylic acid, H_2_O_2_, chilling stress

## Abstract

Grafting with pumpkin rootstock could improve chilling tolerance in watermelon, and salicylic acid (SA) as a signal molecule is involved in regulating plant tolerance to chilling and other abiotic stresses. To clarify the mechanism in pumpkin rootstock-induced systemic acquired acclimation in grafted watermelon under chilling stress, we used self-grafted (*Cl*/*Cl*) and pumpkin rootstock-grafted (*Cl*/*Cm*) watermelon seedlings to study the changes in lipid peroxidation, photosystem II (PSII) activity and antioxidant metabolism, the spatio–temporal response of SA biosynthesis and H_2_O_2_ accumulation to chilling, and the role of H_2_O_2_ signal in SA-induced chilling tolerance in grafted watermelon. The results showed that pumpkin rootstock grafting promoted SA biosynthesis in the watermelon scions. Chilling induced hydrolysis of conjugated SA into free SA in the roots and accumulation of free SA in the leaves in *Cl*/*Cm* plants. Further, pumpkin rootstock grafting induced early response of antioxidant enzyme system in the roots and increased activities of ascorbate peroxidase and glutathione reductase in the leaves, thus maintaining cellular redox homeostasis. Exogenous SA improved while the inhibition of SA biosynthesis reduced chilling tolerance in *Cl*/*Cl* seedlings. The application of diphenyleneiodonium (DPI, inhibitor of NADPH oxidase) and dimethylthiourea (DMTU, H_2_O_2_ scavenger) decreased, while exogenous H_2_O_2_ improved the PSII activity in *Cl/Cl* plants under chilling stress. Additionally, the decrease of the net photosynthetic rate in DMTU- and DPI-pretreated *Cl*/*Cl* plants under chilling conditions could be alleviated by subsequent application of H_2_O_2_ but not SA. In conclusion, pumpkin rootstock grafting induces SA biosynthesis and redistribution in the leaves and roots and participates in the regulation of antioxidant metabolism probably through interaction with the H_2_O_2_ signal, thus improving chilling tolerance in watermelon.

## 1. Introduction

Watermelon (*Citrullus lanatus*) is a warmth-loving plant originating from tropical Africa. It requires a higher temperature in the whole process of growth and development, being not resistant to temperatures below 15 °C. Watermelon plants usually suffer from chilling (0~15 °C) or freezing (<0 °C) stress when grown in the greenhouse in winter and early spring. As a stress factor affecting crop yield and quality, low temperature will cause a series of visible symptoms such as leaf wilting, chlorosis, or necrosis accompanied by many changes in physiological and biochemical cell functions [1]. Grafting, as important agricultural production technology, has been widely used in the production of horticultural crops to overcome soil-borne diseases caused by continuous cropping and improve the adaptability of horticultural crops to abiotic stresses such as low temperature.

Grafting tomatoes onto a cold-tolerant wild species increased the relative growth rate of shoots due to higher root mass ratios at suboptimal (15 °C) air/root zone temperatures [2]. The phytohormones of abscisic acid (ABA) and cytokinins (CTKs) were reported to transport from chilling-tolerant figleaf gourd (*Cucurbita ficifolia*) roots and protect leaf photosynthesis in chilling-sensitive cucumber plants [3]. Li et al. [4] found that under sub-optimal conditions, figleaf gourd rootstock with low-temperature tolerance induced increased expression of stress-responsive genes and activities of antioxidant enzymes, thus improving the photosynthetic efficiency of grafted cucumber plants. For watermelons, the most commonly used grafting rootstocks are pumpkins and gourds. Watermelon (‘Zaojia 8424’) grafted onto cold-tolerant gourds showed higher chlorophyll and proline content and lower malondialdehyde (MDA) content accompanied by enhanced antioxidant activity and higher expression of enzymes related to the Calvin cycle under cold stress [5]. Further, increased accumulation of melatonin, methyl jasmonate (MeJA), and hydrogen peroxide (H_2_O_2_) were observed in pumpkin or figleaf gourd-grafted watermelon plants, and the melatonin-MeJA self-amplifying feedback loop combined with H_2_O_2_ signal demonstrated a novel regulatory mechanism of rootstock-induced cold tolerance in watermelon [6].

As a phenolic phytohormone and signal molecule widely present in higher plants, salicylic acid (SA) affects water metabolism, mineral nutrient absorption, and photosynthesis, and participates in regulating physiological processes such as seed germination, flowering, and ion transmembrane transport [7,8]. In plants, SA biosynthesis has now been fully known to originate from two pathways: the isochorismate synthase (ICS) pathway and the phenylalanine ammonia–lyase (PAL) pathway [9,10,11]. Both are biosynthetic pathways starting in plastids from chorismate and then transferring to cytosol to finally synthesize SA [12]. ICS is the major pathway, contributing to more than 90% of SA biosynthesis involving ICS enzyme and *Enhanced Disease Susceptibility 5* (*EDS5*)-, *avrPphB Susceptible 3* (*PBS3*)-, and *Enhanced Pseudomonas Susceptibility 1* (*EPS1*)-encoded enzymes [9,13]. Additionally, plants utilize the PAL pathway to synthesize a minor fraction (~10%) of SA [12]. Recent studies have found that SA plays a regulatory role in abiotic stresses, and exogenous SA treatment can improve plant tolerance to drought, low/high temperature, salinity, heavy metals, and other stresses [14,15,16,17]. In addition, low temperature induced the increased accumulation of endogenous free and conjugated SA in cucumber and watermelon plants, which was attributed to the increased gene expression and enzyme activities of PAL and benzoic acid 2-hydroxylase (BA2H) [18,19]. Co-inoculation of arbuscular mycorrhizal fungi and the plant growth-promoting rhizobacteria was reported to improve growth and photosynthesis by increasing the activity of PAL and accumulation of phenols and flavonoids in tobacco under drought stress [20]. Moreover, increased phenols content and improved growth were observed in exogenous melatonin-pretreated mallow plants under cadmium stress, which could be due to the induction of PAL activity and an increase in shoot soluble carbohydrates [21]. Glutathione and ascorbic acid in cells are important buffering agents that regulate cell redox homeostasis and prevent redox state imbalance caused by changes in environmental conditions [22]. Interestingly, evidence indicates that SA interplayed with reactive oxygen species (ROS) and glutathione in stressed plants to induce defense responses [23]. However, how endogenous SA responds to chilling stress in grafted watermelon plants and whether SA mediates chilling tolerance of grafted watermelon by changing the cellular redox status has not been illustrated.

In order to clarify the mechanism of pumpkin rootstock grafting in improving chilling tolerance of watermelon, we investigated the spatio–temporal response of chlorophyll fluorescence, membrane lipid peroxidation, antioxidant enzyme activities, cellular redox status, SA biosynthesis, and H_2_O_2_ accumulation to chilling stress in self-grafted and pumpkin rootstock-grafted watermelon plants. Additionally, by using SA biosynthesis inhibitor and H_2_O_2_ inhibitors, we found that the chilling tolerance in pumpkin rootstock-grafted watermelon depended on the interaction between the H_2_O_2_ signal and SA.

## 2. Materials and Methods

### 2.1. Plant Materials and Experimental Design

Watermelon inbred line [*Citrullus lanatus* (Thunb.) Matsum. and Nakai var. *lanatus*] ‘97103’ was taken as scion and ‘Qingyan No.1’ pumpkin was taken as rootstock. Pumpkin rootstock-grafted seedlings (*Cl*/*Cm*) and watermelon ‘97103’ self-grafted seedlings (*Cl*/*Cl*) were obtained using hole insertion grafting method. Seedlings were grown at 28/18 °C (day/night), photoperiod of 12 h/12 h, light intensity of 300 μmol m^−2^ s^−1^, and relative humidity of 70~85%. When the scion grew to four-leaf stage, half of the *Cl*/*Cm* or *Cl*/*Cl* seedlings were treated under 10/5 °C (day/night) as chilling stress, and the other halves were still grown under 28/18 °C (day/night) as control. After 0, 1, 3, 5, and 7 days of low-temperature treatment, leaf chlorophyll fluorescence was measured and leaf and root samples were taken at indicated times, respectively. After freezing with liquid nitrogen, the samples were stored at −80 °C before lipid peroxidation, antioxidant, SA content, PAL activity, and H_2_O_2_ accumulation assays.

To examine the effects of exogenous SA on chilling tolerance of grafted watermelon seedlings, 2/3 of the *Cl*/*Cl* or *Cl*/*Cm* seedlings at the four-leaf stage were pretreated with water. While 1/3 of the *Cl*/*Cl* or *Cl*/*Cm* seedlings were pretreated with 50 µM SA. After 24 h, half of the water-treated and totally SA-treated *Cl*/*Cl* or *Cl*/*Cm* plants were placed in growth chambers at 10/5 °C for 5 days. The remaining water-treated *Cl*/*Cl* or *Cl*/*Cm* plants were maintained in a growth chamber at 28/18 °C to serve as the control. The chlorophyll fluorescence imaging was taken at 0, 1, 3, 5 d after chilling stress. Further, endogenous SA biosynthesis was inhibited by spraying with 50 μM L-α-aminooxy-β-phenylpropionic acid (AOPP), and SA recovery experiment for chilling tolerance in *Cl*/*Cl* seedlings was conducted as previously described [19].

To examine the role of H_2_O_2_ signaling in SA-induced chilling tolerance, firstly, *Cl*/*Cl* seedlings were sprayed with 1 mM H_2_O_2_, 20 µM diphenyleneiodonium (DPI, an NADPH oxidase inhibitor) and 20 mM dimethylthiourea (DMTU, a H_2_O_2_ and ^•^OH scavenger), respectively, prior to chilling stress [24], and leaf chlorophyll fluorescence was measured after 3 days of chilling stress. Secondly, the DPI- and DMTU-pretreated plants were subsequently sprayed with water, H_2_O_2_, or SA before cold treatment, and photosynthetic gas exchange was again determined after 3 days of chilling stress. For all the exogenous spraying treatments, Tween-20 was mixed into each solution and an aliquot of 10 mL was applied per plant using a plastic sprayer.

### 2.2. Analysis of Chlorophyll Fluorescence and Photosynthetic Gas Exchange

Chlorophyll fluorescence at the whole area of the third leaf from the bottom was measured by using Pulse-Amplitude Modulation (PAM) imaging (MAXI; Heinz Walz, Effeltrich, Germany). The seedlings were adapted in the dark for at least 30 min before the measurements were taken. The intensities of the actinic light and saturating light were set at 280 and 4000 μmol m^−2^ s^−1^, respectively. The maximum quantum yield of PSII (*F*v/*F*m) and effective quantum yield of PSII (*Φ*_PSII_) were measured and calculated in accordance with the method described by [25]. *F*v/*F*m = (*F*m − *F*o)/*F*m and *Φ*_PSII_ = (*F’*m − *F*s)/*F’*m. The net photosynthetic rate (*P*n) was measured between 9:00–12:00 in the morning with an open gas exchange system (LI-6400 XT; Li-Cor, Lincoln, NE, USA) on the third leaf of each plant with a CO_2_ concentration of 410 µmol mol^−^^1^, a photosynthetic photon flux density of 300 µmol m^−^^2^ s^−^^1^, a leaf temperature of 25 ± 1.5 °C, and a relative air humidity of 80–90%.

### 2.3. Determination of Lipid Peroxidation and Antioxidant Enzyme Activities

For lipid peroxidation and antioxidant enzyme assays, leaf or root tissues (0.3 g) were ground with a 2 mL ice-cold buffer containing 50 mM phosphate-buffered saline (pH 7.8), 0.2 mM EDTA, 2 mM L-ascorbic acid, and 2% (*w*/*v*) polyvinylpyrrolidone. Homogenates were centrifuged at 12,000× *g* for 20 min, and the resulting supernatants were used to determine the MDA content and enzyme activities. The samples for MDA determination were mixed with 10% trichloroacetic acid that contained 0.65% 2-thiobarbituric acid (TBA) and heated at 95 °C for 25 min. Then, MDA equivalents were corrected for the non-MDA compounds by subtracting the absorbance at 532 nm of a TBA-less solution that contained the plant extract [26]. Catalase (CAT) activity was measured as a decline in *A*_240_ in accordance with the method described by Patra et al. [27]. Peroxidase (POD) activity was measured as an increase in *A*_470_ by using guaiacol as a substrate [28]. Ascorbate peroxidase (APX) activity was measured as a decrease in *A*_290_, as described by [29]. Glutathione reductase (GR) activity was measured based on the decrease of NADPH at *A*_340_ according to Halliwell and Foyer [30]. Total antioxidant capacity (T-AOC) was determined with the ability to reduce Fe^3+^ to Fe^2+^, as previously described [19]. All spectrophotometric analyses were conducted on an Infinite M200 PRO Multi-Detection Microplate Reader (Tecan, Männedorf, Zürich, Switzerland).

### 2.4. Measurements of Glutathione and Ascorbate Contents

For the measurement of reduced glutathione (GSH) and oxidized glutathione (GSSG), plant leaf tissue (0.3 g) was homogenized in 2 mL of 6% metaphosphoric acid containing 2 mM EDTA and centrifuged at 4 °C for 10 min at 12,000× *g*. After neutralization with 0.5 M phosphate buffer (pH 7.5), 0.1 ml of the supernatant was added to a reaction mixture containing 0.2 mM NADPH, 100 mM phosphate buffer (pH 7.5), 5 mM EDTA, and 0.6 mM 5,5′-dithio-bis (2-nitrobenzoic acid). The reaction was initiated by adding 3 U of GR and was monitored by measuring the changes in absorbance at 412 nm for 1min. For the GSSG assay, GSH was masked by the addition of 40 μL of 2-vinylpyridine to the neutralized supernatant, whereas 40 μL of water was added for the total glutathione assay. The GSH concentration was obtained by subtracting the GSSG concentration from the total concentration [31].

Reduced (AsA) and oxidized (DHA) forms of ascorbate were measured following Law et al. [32]. The total AsA was determined by initially incubating the extract for 50 min with 200 mM phosphate buffer solution (pH 7.4) and 1.5 mM dithiothreitol (DTT) to reduce all DHA to AsA. After incubation, 200 µL of 0.5% (*w*/*v*) N-ethylmaleimide (NEM) was added to remove excess DTT. AsA was analyzed in a similar manner except that 400 µL deionized H_2_O was substituted for DTT and NEM. Color was developed in both series of reaction mixtures (total and reduced ascorbate) with the addition of 400 µL 10% (*w*/*v*) trichloroacetic acid, 400 µL 44% *ο*-phosphoric acid, 4% α′-dipyridyl in 70% ethanol, and 200 µL 3% (*w*/*v*) FeCl_3_. The reaction mixtures were then incubated at 40 °C for 40 min in a water bath and the absorbance was recorded at 525 nm. The DHA concentration was obtained by subtracting the AsA concentration from the total concentration.

### 2.5. Measurements of SA Content and PAL Activity

Free and conjugated SA measurements in leaf and root tissues were conducted using a rapid biosensor-based method, as described by DeFraia et al. [33]. Leaf tissues were ground in liquid nitrogen and then left at room temperature for 5 min. Acetate buffer (0.1 M, pH 5.6) was added at a ratio of 2.5 μL/mg tissue at room temperature before samples were mixed and centrifuged for 15 min at 16,000× *g*. Half (100 μL) of the supernatant was stored on ice for free SA measurement, and the other half was incubated at 37 °C for 90 min with 4 U of β-glucosidase (3.2.1.21, Sigma-Aldrich, St. Louis, MO, USA) for conjugated SA measurement. An overnight biosensor culture of Acinetobacter sp. ADPWH_lux was diluted in 37 °C LB (1:20) and grown for ~3 h at 200 rpm to an OD600 of 0.4. Up to 20 μL of crude extract that was stored at room temperature (20–22 °C) was added to 60 μL of LB and 50 μL of biosensor culture in a black 96-well cell culture plate. The plate was incubated at 37 °C for 1 h without shaking before luminescence was read on an Infinite M200 Pro Multi-Detection Microplate Reader (Tecan, Männedorf, Zürich, Switzerland).

For the PAL activity, leaf or root tissues (0.3 g) were ground in liquid nitrogen and then added with 1.5 mL of ice-cold buffer containing 50 mM Tris-HCl (pH 8.5), 5 mM EDTA, 15 mM *β*-mercaptoethanol, 1 mM 4-(2-Aminoethyl) benzenesulfonyl fluoride hydrochloride (AEBSF), and 0.15% (*w*/*v*) polyvinylpyrrolidone (PVP). Homogenates were centrifuged at 12,000× *g* for 20 min at 4 °C, and the resulting supernatants were used to determine PAL activity on the basis of the formation of trans-cinnamic acid monitored at 290 nm [34].

### 2.6. Determination of Electrolyte Leakage

Electrolyte leakage of the third fully expanded leaves was measured after chilling stress according to Hong et al. [35] with minor modifications. Briefly, 0.1 g of leaf samples were cut into 1-centimeter^2^ fragments, rinsed with deionized water, and then shaken for 3 h at 22 °C. The electrolyte leakage was calculated by the percentage of conductivity before (EL1) and after (EL2) boiling of the leaf fragments. Electrolyte leakage (%) = EL1/EL2 × 100.

### 2.7. Determination of H_2_O_2_ Content

To determine the H_2_O_2_ content, 0.3-gram leaf tissues were sampled and ground in 3 mL of 1 M HClO_4_. Then, the mixture was transferred to a 10-mililiter plastic tube. The homogenate was centrifuged at 6000× *g* for 5 min at 4 °C and the supernatant was collected, adjusted to pH 6.0 with 4 M KOH, and centrifuged at 110 g for 1 min at 4 °C. The supernatant was placed onto a AG 1-X8 prepacked column (Bio-Rad, Hercules, CA, USA), and H_2_O_2_ was eluted with 4 mL of double-distilled H_2_O. The sample (800 μL) was mixed with 400 μL of reaction buffer containing 4 mM 2,2′-azino-di (3-ethylbenzthiazoline-6-sulfonic acid) and 100 mM potassium acetate at pH 4.4, 400 μL of deionized water, and 0.25 U of horseradish peroxidase. The H_2_O_2_ content was measured at OD_412_ [36].

### 2.8. Statistical Analysis

The experiment involved a completely randomized block design with four replicates, and each replicate consisted of 10 grafted watermelon seedlings. Statistical analysis was performed using the SAS statistical package. The differences between the treatment means were separated using Tukey’s test at a significance level of *p* < 0.05.

## 3. Results

### 3.1. Pumpkin Rootstock Alleviated the Oxidative Damage Caused by Chilling Stress in Grafted Watermelon Seedlings

**Figure 1:**
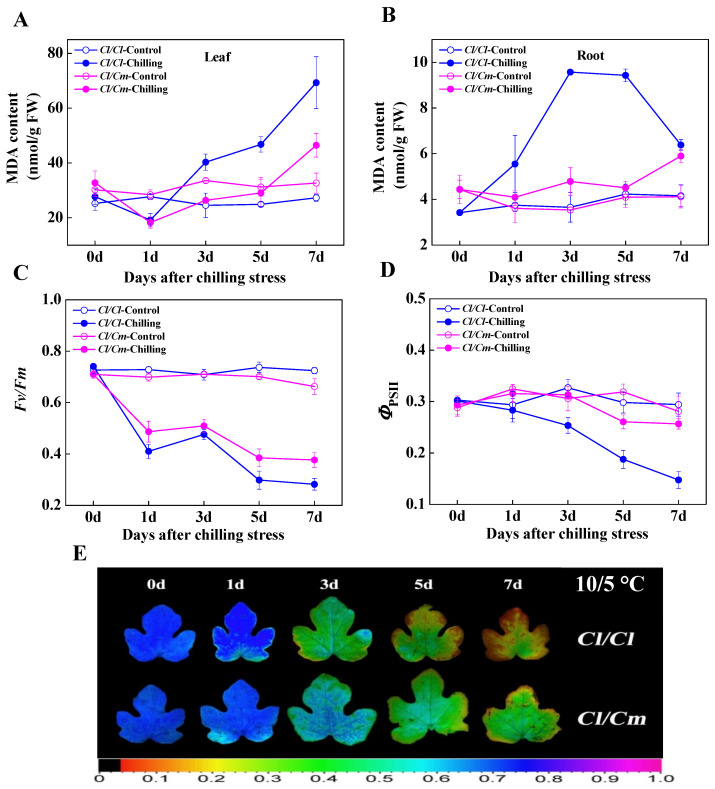
Pumpkin rootstock-induced chilling tolerance in grafted watermelon plants. (**A**) Changes in malondialdehyde (MDA) content in the leaves under chilling stress. (**B**) Changes in MDA content in the roots under chilling stress. (**C**) Average values of the maximum quantum yield of PSII (*F*v/*F*m). (**D**) Average values of the effective quantum yield of PSII (*Φ*_PSII_). (**E**) Images of *F*v/*F*m under chilling stress. Leaf or root samples were collected at indicated times under control (28/18 °C) and chilling (10/5 °C) conditions. *Cl*/*Cl*, self-grafted watermelon plants; *Cl/Cm*, pumpkin rootstock-grafted watermelon plants. The data are the means of four replicates with SEs. The color gradient of the images in *F*v/*F*m provided at the bottom of Figure 1E ranged from 0 (black) to 1.0 (purple).

At normal temperature (28/18 °C), the MDA content in leaves and roots of pumpkin rootstock-grafted (*Cl*/*Cm*) seedlings was similar to that of watermelon self-grafted (*Cl*/*Cl*) seedlings (Figure 1A,B). However, MDA content in leaves and roots of *Cl*/*Cl* seedlings increased significantly after chilling stress, while the content in *Cl*/*Cm* seedlings showed no significant accumulation after five days of chilling treatment. At seven days of chilling stress, the MDA content in leaves and roots of *Cl*/*Cl* seedlings increased by 154.55% and 67.50%, respectively compared with the control. By contrast, 56.67% increase in leaves and 47.50% increase in roots in MDA content were observed in *Cl*/*Cm* plants in comparison with control (Figure 1A,B). Similarly, the *F*v/*F*m and *Φ*_PSII_, two popular metrics for quantifying photo-oxidative stress, in the leaves of *Cl*/*Cl* and *Cl*/*Cm* seedlings showed no significant differences and remained relatively stable at normal temperature (Figure 1C,D). The *F*v/*F*m decreased promptly in *Cl*/*Cl* seedlings after one day of chilling stress, while that in *Cl*/*Cm* seedlings decreased more sluggishly in chilling-stressed plants compared with control (Figure 1C). As shown in Figure 1E, the image of *F*v/*F*m showed more serious photo-oxidative damage in *Cl*/*Cl* leaves compared with *Cl*/*Cm* leaves after seven days of chilling stress. Additionally, the *Φ*_PSII_ showed constant decrease in *Cl*/*Cl* seedlings within seven days of chilling stress, while that in *Cl/Cm* seedlings decreased slightly in comparison with control (Figure 1D). These results indicated that pumpkin rootstock alleviated the oxidative damage of chilling stress in grafted watermelon seedlings.

### 3.2. Chilling-Induced Changes in Antioxidant Enzyme System and Cellular Redox Homeostasis in Grafted Watermelon Seedlings

**Figure 2:**
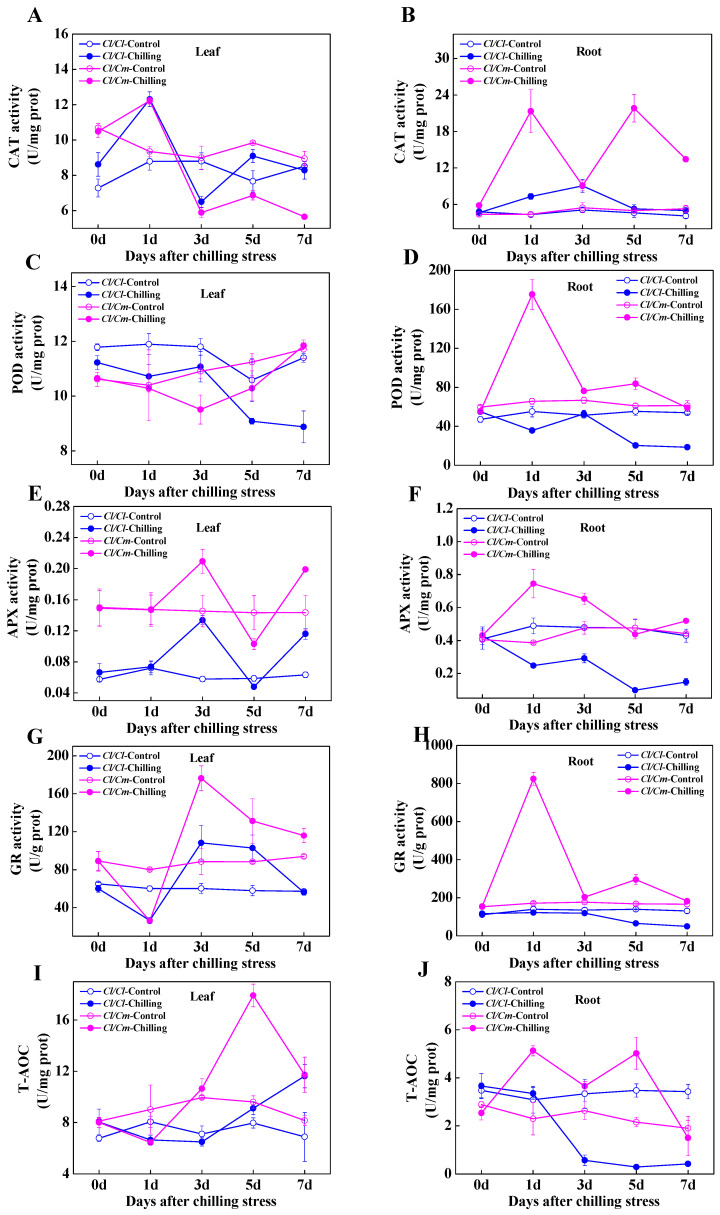
Dynamic changes in the activities of antioxidant enzyme system in grafted watermelon plants in response to chilling stress. (**A**) Catalase (CAT), (**C**) Peroxidase (POD), (**E**) Ascorbate peroxidase (APX), and (**G**) Glutathione reductase (GR) activities in the leaves. (**B**) CAT, (**D**) POD, (**F**) APX, and (**H**) GR activities in the roots. (**I**) Total antioxidant capacity (T-AOC) in the leaves. (**J**) T-AOC in the roots. Leaf or root samples were collected at indicated times under control (28/18 °C) and chilling (10/5 °C) conditions. *Cl*/*Cl*, self-grafted watermelon plants; *Cl*/*Cm*, pumpkin rootstock-grafted watermelon plants. The data are the means of four replicates with SEs.

To investigate the antioxidative response to chilling stress in *Cl*/*Cl* and *Cl*/*Cm* seedlings, we examined the changes in activities of four antioxidant enzymes and total antioxidant capacity (T-AOC). The activities of CAT, POD, APX, GR, and T-AOC of the roots increased significantly in *Cl*/*Cm* seedlings, peaked at one day after chilling stress. However, the activities of POD, APX, GR, and T-AOC, except for CAT decreased gradually with chilling treatment in the roots of *Cl*/*Cl* seedlings (Figure 2B,D,F,H,J). After 1 d of chilling treatment, the CAT activity in leaves of *Cl*/*Cl* and *Cl*/*Cm* seedlings increased significantly, then decreased under the level of control after three days of chilling stress (Figure 2A). Interestingly, the activities of APX and GR increased significantly and peaked at three days of chilling treatment in the leaves of both *Cl*/*Cl* and *Cl*/*Cm* seedlings, with the highest level in chilling-stressed *Cl*/*Cm* leaves (Figure 2E,G). On the contrary, POD activity in the leaves of *Cl*/*Cl* and *Cl*/*Cm* plants showed lower levels of control within the seven days of chilling stress (Figure 2C). The T-AOC reached the highest level in leaves of *Cl*/*Cm* plants after five days of chilling treatment, then decreased to a similar level of that in leaves of *Cl*/*Cl* plants after seven days of chilling treatment (Figure 2I).

**Figure 3:**
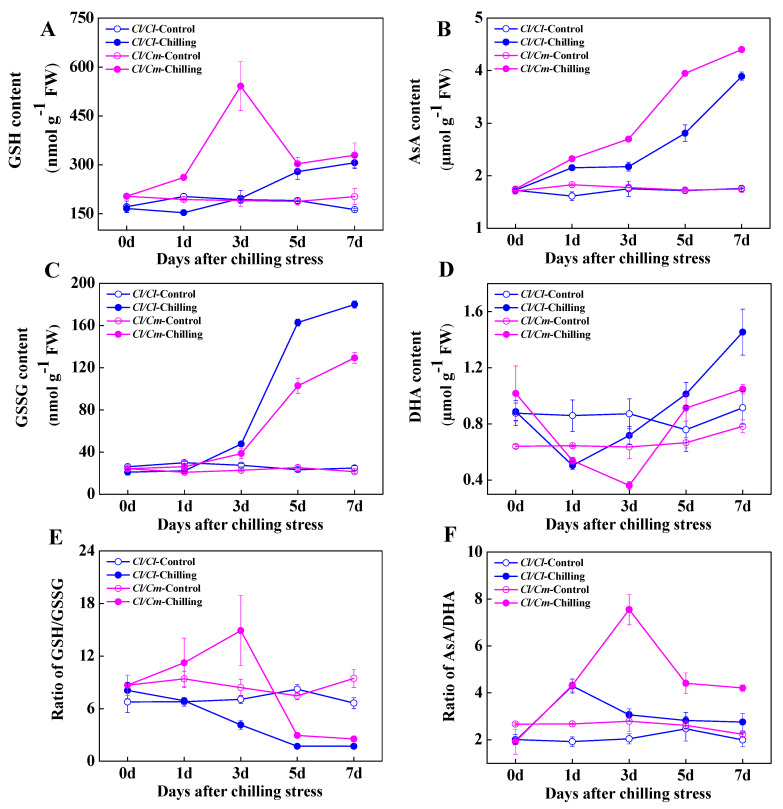
Chilling-induced changes in glutathione and ascorbate homeostasis in grafted watermelon leaves. (**A**) Reduced (GSH) and (**C**) Oxidized glutathione (GSSG) content. (**B**) Reduced (AsA) and (**D**) Oxidized ascorbate content. (**E**) The ratio of GSH/GSSG content. (**F**) The ratio of AsA/DHA content. Leaf samples were collected at indicated times under control (28/18 °C) and chilling (10/5 °C) conditions. *Cl*/*Cl*, self-grafted watermelon plants; *Cl*/*Cm*, pumpkin rootstock-grafted watermelon plants. The data are the means of four replicates with SEs. rootstock induced SA biosynthesis in the leaves and roots of grafted watermelon seedlings, and chilling induced hydrolysis of conjugated SA into free SA in the roots combined with increased accumulation of free SA in the leaves of *Cl*/*Cm* plants probably serve to improve chilling tolerance. In addition, the activity of PAL in the leaves of *Cl*/*Cm* plants was significantly higher than that in *Cl*/*Cl* plants under normal temperature (Figure 5A). Chilling induced significant increase in PAL activity in the leaves and roots of *Cl*/*Cl* and *Cl*/*Cm* plants, which showed that PAL activity in the roots of *Cl*/*Cm* plants peaked at 3 d of chilling stress in comparison with control (Figure 5).

Glutathione and ascorbate are important non-enzymatic antioxidants and play pivotal roles in cellular redox homeostasis. In the present study, no significant differences were observed in the content of GSH and GSSG, and GSH/GSSG ratio between *Cl*/*Cl* and *Cl*/*Cm* leaves at normal temperature (Figure 3A,C,E). Chilling induced a significant increase in the GSH content and peaked at three days in stressed *Cl*/*Cm* plants compared with control. However, the GSH content in *Cl*/*Cl* leaves showed minor changes in response to chilling stress (Figure 3A). Importantly, the GSSG was shown to accumulate after three days of chilling stress in both *Cl*/*Cl* and *Cl*/*Cm* leaves with a higher level in *Cl*/*Cl* plants (Figure 3C). Therefore, the ratio of GSH/GSSG in leaves of *Cl*/*Cm* plants increased significantly and peaked at three days of chilling treatment. While the GSH/GSSG ratio in *Cl*/*Cl* leaves decreased as a result of the increased accumulation of GSSG under chilling stress (Figure 3E). Moreover, the AsA content continuously increased in both *Cl*/*Cl* and *Cl*/*Cm* leaves within seven days of chilling stress, with a higher level in *Cl*/*Cm* plants (Figure 3B). On the contrary, chilling induced a significant decrease of DHA content at one day or three days of chilling stress in *Cl*/*Cl* and *Cl*/*Cm* leaves, respectively (Figure 3D). As a result, a significant increase in the ratio of AsA/DHA in *Cl*/*Cl* and *Cl*/*Cm* leaves was observed at one day or three days of chilling stress, respectively (Figure 3F). These results suggested that pumpkin rootstock induced early response of antioxidant enzyme system in the roots under chilling stress, and the subsequently increased activities of the antioxidant enzyme system and changes in cellular redox status in the leaves jointly regulated chilling tolerance of grafted watermelon.

### 3.3. SA Was Involved in the Regulation of Chilling Tolerance in Pumpkin Rootstock-Grafterd Watermelon Seedlings

**Figure 4:**
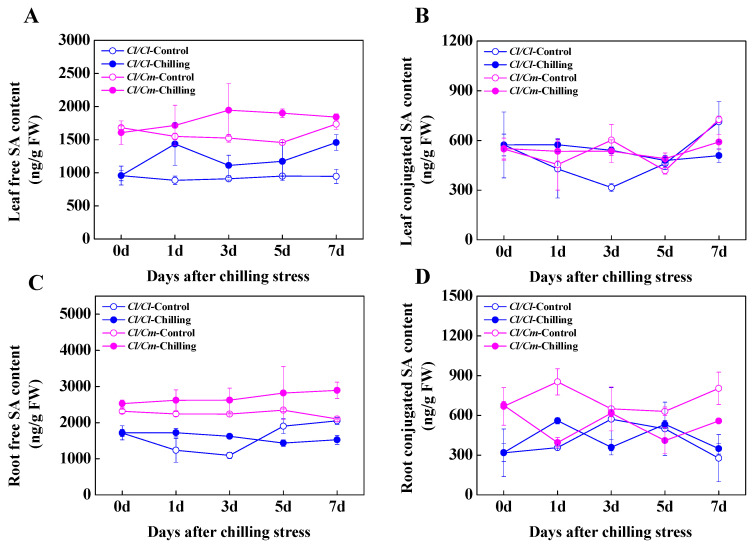
The spatio–temporal response of salicylic acid (SA) content to chilling stress in grafted watermelon plants. (**A**) Free and (**B**) Conjugated SA content in the leaves. (**C**) Free and (**D**) Conjugated SA content in the roots. Leaf or root samples were collected at indicated times under control (28/18 °C) and chilling (10/5 °C) conditions. *Cl*/*Cl*, self-grafted watermelon plants; *Cl*/*Cm*, pumpkin rootstock-grafted watermelon plants. The data are the means of four replicates with SEs.

**Figure 5:**
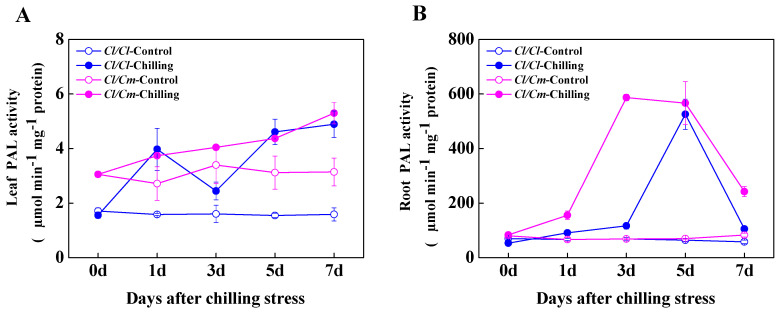
The time-course response of phenylalanine ammonia-lyase (PAL) activity to chilling stress in grafted watermelon plants. (**A**) PAL activity in the leaves. (**B**) PAL activity in the roots. Leaf or root samples were collected at indicated times under control (28/18 °C) and chilling (10/5 °C) conditions. *Cl*/*Cl*, self-grafted watermelon plants; *Cl*/*Cm*, pumpkin rootstock-grafted watermelon plants. The data are the means of four replicates with SEs.

To determine the role of SA in chilling stress response in grafted watermelon plants, we examined the free and conjugated SA contents in the leaves and roots of *Cl*/*Cl* and *Cl*/*Cm* seedlings during chilling stress (Figure 4). At normal temperature, the content of free and conjugated SA in leaves and roots of *Cl*/*Cm* plants was significantly higher than that of *Cl*/*Cl* plants (Figure 4A,C). The content of free and conjugated SA in the roots of *Cl*/*Cl* plants increased slightly and then decreased under the level of control after seven days of chilling stress, while the free and conjugated SA in the roots of *Cl*/*Cm* plants showed increased and decreased accumulation in response to chilling stress, respectively (Figure 4C,D). Under chilling conditions, the content of free SA in leaves of both *Cl*/*Cm* and *Cl*/*Cl* plants continued to increase, while the content of conjugated SA did not change significantly compared with the control (Figure 4A,B). These results indicated that pumpkin rootstock induced SA biosynthesis in the leaves and roots of grafted watermelon seedlings, and chilling induced hydrolysis of conjugated SA into free SA in the roots combined with increased accumulation of free SA in the leaves of *Cl*/*Cm* plants, probably serve to improve chilling tolerance. In addition, the activity of PAL in the leaves of *Cl*/*Cm* plants was significantly higher than that in *Cl*/*Cl* plants under normal temperature (Figure 5A). Chilling induced a significant increase in PAL activity in the leaves and roots of *Cl/Cl* and *Cl*/*Cm* plants, which showed that PAL activity in the roots of *Cl*/*Cm* plants peaked at three days of chilling stress in comparison with control (Figure 5).

**Figure 6:**
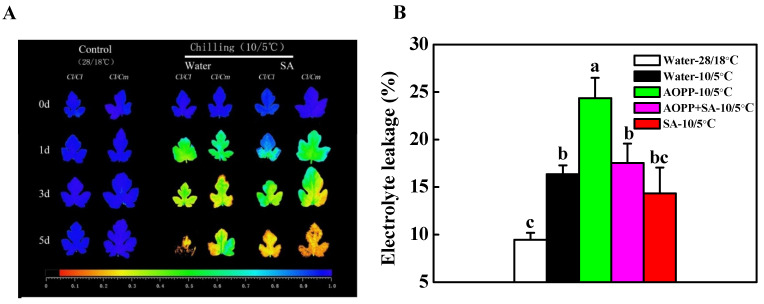
The role of SA-induced chilling tolerance in grafted watermelon plants. (**A**) Images of *F*v/*F*m in water or SA-pretreated *Cl*/*Cl* and *Cl*/*Cm* plants under chilling stress. Leaf samples were collected at indicated times under control (28/18 °C) and chilling (10/5 °C) conditions. (**B**) Electrolyte leakage in the leaves of water, AOPP, AOPP+SA, and SA-pretreated *Cl*/*Cl* plants after 3 days of chilling stress. AOPP, L-α-aminooxy-β-phenyl propionic acid. Both SA and AOPP were treated at 50 µM. The data are the means of four replicates with SEs. Different letters indicate significant differences according to Tukey’s test (*p* < 0.05). *Cl*/*Cl*, self-grafted watermelon plants; *Cl*/*Cm*, pumpkin rootstock-grafted watermelon plants. The color gradient of the images in *F*v/*F*m provided at the bottom of Figure 6A ranged from 0 (black) to 1.0 (purple).

The *F*v/*F*m and electrolyte leakage are commonly used indicators to evaluate chilling tolerance in plants. Here, we analyzed the SA-induced changes in *F*v/*F*m and electrolyte leakage after chilling stress (Figure 6). Water and 50 μM of SA were pretreated before the *Cl*/*Cl* and *Cl*/*Cm* seedlings were exposed at 10/5 °C for five days. As shown in Figure 6A, the images of *F*v/*F*m in the leaves of *Cl*/*Cl* and *Cl*/*Cm* seedlings exhibited no significant differences when grown at normal temperature. Chilling induced a substantial decrease in *F*v/*F*m in water-treated *Cl*/*Cl* plants, while SA pretreatment alleviated the PSII damage in *Cl*/*Cl* plants as indicated by better performance of *F*v/*F*m imaging. Furthermore, the mitigation of PSII damage in pumpkin rootstock-grafted *Cl*/*Cm* plants under chilling stress was compromised in SA-pretreated *Cl*/*Cl* and *Cl*/*Cm* plants, implying an important role of SA in pumpkin rootstock-induced chilling tolerance. The electrolyte leakage in self-grafted *Cl*/*Cl* leaves in response to chilling stress was also determined by altering the cellular SA levels (Figure 6B). Chilling induced 172.8% increase in the electrolyte leakage in water-treated *Cl*/*Cl* plants, while SA pretreatment alleviated the electrolyte leakage in chilling-stressed *Cl*/*Cl* leaves. Exogenous treatment of 50 μM AOPP (inhibitor of SA biosynthesis) induced 257.4% increase in the electrolyte leakage in chilling-stressed *Cl*/*Cl* plants as compared with the control. However, the increase of electrolyte leakage in AOPP-treated leaves was compromised by the subsequent application of SA under chilling stress (Figure 6B).

### 3.4. The H_2_O_2_ Signal Was Involved in SA-Induced Chilling Tolerance in Grafted Watermelon Seedlings

**Figure 7:**
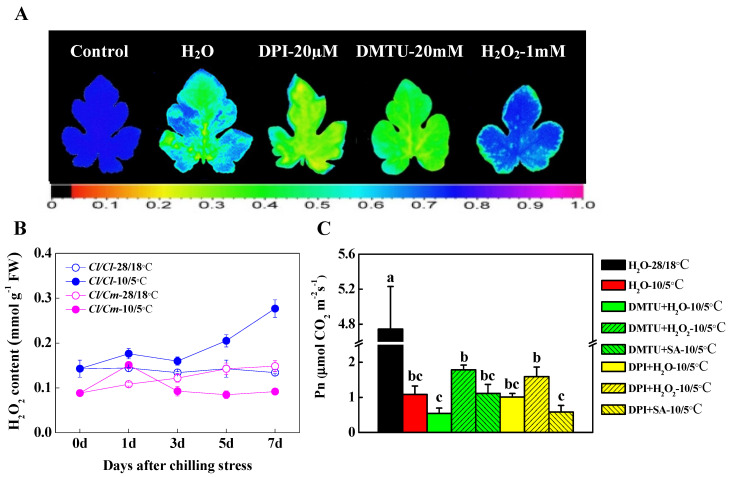
H_2_O_2_ signal mediates SA-induced chilling tolerance in grafted watermelon plants. (**A**) Images of *F*v/*F*m in water, DPI, DMTU, and H_2_O_2_-pretreated *Cl*/*Cl* plants under chilling stress. Plants were pretreated with 20 µM DPI or 20 mM DMTU for 8 h, while water and H_2_O_2_ (1 mM) were pretreated for 24 h. Leaf samples were collected after 3 days of chilling (10/5 °C) stress for chlorophyll fluorescence analysis. (**B**) H_2_O_2_ accumulation in response to chilling stress. (**C**) Changes in the net photosynthetic rate (*P*n) in the leaves of *Cl*/*Cl* plants under chilling stress. Plants were pretreated with 20mM DMTU or 20 µM DPI for 8 h, and then treated with water, H_2_O_2_ (1 mM), and SA (50 µM), respectively. The *P*n was determined after 3 days of chilling stress. The data are the means of four replicates with SEs. Different letters indicate significant differences according to Tukey’s test (*p* < 0.05). *Cl*/*Cl*, self-grafted watermelon plants; *Cl*/*Cm*, pumpkin rootstock-grafted watermelon plants. The color gradient of the images in *F*v/*F*m provided at the bottom of Figure 7A ranged from 0 (black) to 1.0 (purple).

Cellular ROS signaling plays important roles in the acclimation of plants to various abiotic stresses. We used different concentrations of DPI (inhibitor of NADPH oxidase), DMTU (H_2_O_2_ scavenger), and exogenous H_2_O_2_ to examine the role of H_2_O_2_-induced chilling tolerance in self-grafted *Cl*/*Cl* plants (data not shown). Our results showed that 20 μM DPI and 20 mM DMTU significantly inhibited the PSII activity and increased the sensitivity of *Cl*/*Cl* seedlings to chilling stress as indicated by lower values of *F*v/*F*m imaging compared with water-treated plants (Figure 7A). On the contrary, exogenous spraying of different concentrations of H_2_O_2_ effectively improved the PSII activity in chilling-stressed *Cl*/*Cl* seedlings (data not shown), and the optimal H_2_O_2_ concentration to protect the leaves from photo-oxidative damage was 1mM (Figure 7A). These results suggested that H_2_O_2_ could reduce PSII damage in watermelon leaves under chilling stress, and thus enhances the chilling tolerance of watermelon. We also detected the H_2_O_2_ content in response to chilling stress in grafted watermelon leaves (Figure 7B). The results showed that the H_2_O_2_ content in *Cl*/*Cl* and *Cl*/*Cm* plants remained stable within seven days at normal temperature (28/18 °C). However, H_2_O_2_ accumulation in *Cl*/*Cl* seedlings was continuously induced by chilling stress, while that in *Cl*/*Cm* seedlings peaked at one day of chilling treatment, and then began to decrease under the level of control within seven days of chilling stress (Figure 7B). Therefore, we speculated that H_2_O_2_ signal was likely involved in the early response of grafted watermelon to chilling stress and played a role in the downstream of SA to regulate chilling tolerance in pumpkin rootstock-grafted watermelon plants.

We used self-grafted *Cl*/*Cl* seedlings as materials to study the role of H_2_O_2_ in SA-induced chilling tolerance in grafted watermelon plants (Figure 7C). The *P*n in *Cl*/*Cl* leaves decreased significantly under chilling stress in comparison with control (28/18 °C). DMTU and DPI pre-treatment further reduced the *P*n in chilling-stressed *Cl*/*Cl* leaves, and subsequent exogenous H_2_O_2_ treatment could effectively alleviate the decrease of *P*n under chilling stress. Importantly, the role of SA-induced increase in *P*n under chilling conditions was eliminated in DMTU and DPI pre-treated *Cl*/*Cl* plants, respectively (Figure 7C). These results suggested that the H_2_O_2_ signal was involved in SA-regulated chilling tolerance in pumpkin rootstock-grafted watermelon plants.

## 4. Discussion

### 4.1. SA Biosynthesis Participates in Chilling Stress Response in Grafted Watermelon Plants

SA in plants exists in two main forms: its active free form and its inactive vacuolar storage form, including SA glucoside (SAG) and SA glucose ester (SGE). Conjugated SAG and SGE accumulate in the cell vacuoles in large quantities and can form active, usable forms by hydrolysis [37]. Promoted SA biosynthesis due to pathogen attack played important roles in the regulation of defense response in *Arabidopsis*, tobacco, and citrus fruit [38,39,40]. Additionally, SA functions as a signal of several types of abiotic stresses such as high light exposure, salinity, drought, and low temperature [16,41,42,43]. Here, our results demonstrated that chilling induced a significant increase in free SA in both the leaves and roots of *Cl*/*Cm* plants (Figure 4A,C). Similarly, higher SA accumulation was observed in the leaves, roots, and xylem sap of pumpkin rootstock-grafted than self-grafted cucumber plants due to increased expression of *PAL*, *ICS*, and *SABP2* genes involved in SA biosynthesis and activity of PAL under chilling stress [44]. Furthermore, our previous iTRAQ-based quantitative proteomic study showed a more significant accumulation of PAL protein (Cla008727) in pumpkin rootstock-grafted than self-grafted watermelon plants after exposure to chilling for 48 h [45]. The virus-induced gene silencing of *PAL* in cotton plants showed reduced levels of both free SA and SAG content, suggesting that the SA biosynthesis is critically dependent on the PAL pathway [46]. Many chemical modifications of SA can occur in cells, and glucose conjugation at the hydroxyl group of SA leads to the biosynthesis of inactive SAG which is stored in the vacuolar [47]. In the present study, a significant decrease in the conjugated SA content was shown in the roots of *Cl*/*Cm* plants (Figure 4D), implying the possible formation of active free SA from hydrolyzed SAG in the roots under chilling conditions. Several studies support the notion that both *N*-hydroxy-pipecolic acid and SA are mobile between local and systemic tissue in *Arabidopsis* and tobacco for systemic acquired resistance (SAR) [48,49,50,51]. Accordingly, chilling-stimulated free SA accumulation in the watermelon leaves probably came from the transport of SA from the pumpkin rootstock (Figure 4A,C). These results thus suggest a potential role of SA biosynthesis in the systemic regulation of chilling tolerance in grafted watermelon plants at transcriptional, translational, and subcellular levels.

### 4.2. Differential Response of Antioxidant Enzyme System and Cellular Redox Homeostasis Synergistically Function in Pumpkin Rootstock-Induced Chilling Tolerance in Watermelon

Several types of ROS including Superoxide (O_2_^•−^), hydroxyl (^•^OH), singlet oxygen (^1^O_2_), and H_2_O_2_ are important for plants and play a dual role under various abiotic stresses; a small amount of those acts as a signal for inducing stress responses, while excess generation of those causes oxidative damage to membranes, proteins, DNA, RNA, and even the whole cell [52]. The plant antioxidant defense system comprises enzymatic and non-enzymatic antioxidants in different subcellular localization. Superoxide dismutase (SOD), CAT, POD, APX, monodehydroascorbate reductase (MDHAR), dehydroascorbate reductase (DHAR), GR, and glutathione peroxidase (GPX) are well known antioxidant enzymes, while AsA, GSH, carotenoids, tocopherols, flavonoids, etc. are some commonly known non-enzymatic antioxidants [53]. The AsA-GSH cycle comprises AsA, GSH, APX, MDHAR, DHAR, and GR, which play a vital role in detoxifying ROS. Our present study showed remarkably increased activities of CAT, POD, APX, and GR after 1 day of chilling stress in the roots of *Cl*/*Cm* plants (Figure 2B,D,F,H), suggesting an early response of the antioxidant enzyme system in the pumpkin roots to chilling stress. AsA and GSH are strong antioxidants, but the maintenance of their redox homeostasis is important in conferring stress tolerance in plants, which largely depends on the activities of APX, MDHAR, DHAR, and GR involved in the AsA-GSH cycle [54,55]. Here, it is obvious that chilling induced a substantial increase in the ratios of GSH/GSSG and AsA/DHA after three days of chilling stress in *Cl/Cm* leaves, which was mainly attributed to the increase of GSH content and decrease of DHA content, respectively (Figure 3). The antioxidant enzymes usually showed differential responses in tolerant and sensitive varieties due to cold stress. Javadian et al. [56] showed significant low temperature-induced elevation in activities of CAT and POD in leaves of winter cultivar rather than in spring cultivar in wheat. Differential responses of the activities in SOD, CAT, POD, and APX were also reported in four cultivars of banana, and higher cold tolerance may correlate with the long-term cold adaptation of the antioxidative enzymes such as SOD, POD, and APX that alleviate oxidative stress caused by low temperature [57]. Our present results indicated significantly decreased POD activity in the leaves of chilling-stressed *Cl*/*Cl* and *Cl*/*Cm* plants compared with the control, which could be attributed to the reduced accumulation of POD proteins (Cla002251, Cla003190, Cla014013) as reported in our previous study [45]. However, the specifically increased activities of APX and GR after three days of chilling stress in *Cl*/*Cm* leaves suggest an important role of the AsA-GSH cycle in pumpkin rootstock-induced chilling tolerance in watermelon.

### 4.3. H_2_O_2_ Signal Mediates the Regulation of SA on Chilling Tolerance of Grafted Watermelon

H_2_O_2_ has emerged as a signaling molecule in plants, and its role in early signaling events initiated by environmental stimuli is well established [58,59]. A prominent source of H_2_O_2_ production in the apoplast is *Respiratory Burst Oxidase Homologues* (*RBOHs*)-encoded NADPH oxidases, which use electrons from cytosolic NADPH to reduce oxygen to O_2_^−^ in the apoplast [59]. Here, our results exhibited significantly increased H_2_O_2_ accumulation at one day of chilling stress in *Cl*/*Cm* leaves (Figure 7B), which implies early H_2_O_2_ signaling in response to chilling stress. In cases of mechanical wounding, excessive light, drought, low/high temperature, and salt stress, the H_2_O_2_ bursts are mainly produced via the NADPH oxidase pathway, resulting in the activation of the antioxidant system containing SOD, CAT, APX, and GR, thus alleviating the oxidative damage on PSII activity and photosynthesis [60,61]. Many phytohormones like auxin, brassinosteroids, gibberellins, ABA, ethylene, strigolactones, jasmonic acid, and also SA generate ROS as part of the mechanism that regulates plant growth and development and stress response [62]. The crosstalk of phytohormones in response to abiotic stresses was reported to induce antioxidant defense via distinguished pathways [63]. In *Arabidopsis*, a spatial–temporal interaction of the ROS wave with ABA accumulation in systemic tissues mediates the systemic acquired acclimation (SAA) of plants to heat stress [64]. It is emphasized here that in the present study, the inhibition of H_2_O_2_ by application of DMTU and DPI increased, while exogenous H_2_O_2_ reduced the sensitivity to chilling stress in *Cl*/*Cl* plants (Figure 7A), suggesting a positive role of H_2_O_2_ in the chilling tolerance of grafted watermelon plants. A few studies reported that the SA levels increased upon heat or cold stress in plants, which were shown to improve the photosynthetic capacity by protecting the PSII complex from higher levels of ROS [18,65,66]. Additionally, exogenous application of SA enhanced heat or cold tolerance through activation of antioxidant enzymes such as SOD, CAT, POD, APX, and GR in tomato and watermelon plants [67,68]. In this study, the decrease in *P*n in DMTU and DPI-pretreated *Cl/Cl* plants under chilling stress was alleviated by subsequent H_2_O_2_ treatment but not SA (Figure 7C), indicating that SA-induced chilling tolerance in grafted watermelon plants is dependent on the H_2_O_2_ signal. Thus, a spatial–temporal interaction of the SA accumulation with H_2_O_2_ signal in the distant shoot may mediate the SAA of grafted watermelon plants to chilling stress.

## 5. Conclusions

Overall, we conclude that after a grafted watermelon plant is subjected to chilling stress, the pumpkin root may transmit SA signal to the watermelon shoot, thereby enhancing the activities of APX and GR and modulating the glutathione and ascorbate homeostasis through interaction with H_2_O_2_ signaling, thus improving the photosynthetic efficiency under chilling stress. In the future, we need to further study how the SA interacts with H_2_O_2_ signal in response to chilling stress in plants, and the transcriptome and metabolome analysis in watermelon or pumpkin with varied chilling sensitivity could shed light on the link between phytohormones and antioxidant system in response to chilling stress.

## Data Availability

Data is contained within the article.
